# A Case of Fasciitis Following SARS-CoV-2 mRNA Vaccination: Case Review and Literature Overview

**DOI:** 10.7759/cureus.94531

**Published:** 2025-10-14

**Authors:** Tomoya Harada, Genki Inui, Hiroki Kohno, Ryoko Kimura, Akira Yamasaki

**Affiliations:** 1 Division of Respiratory Medicine and Rheumatology, Department of Multidisciplinary Internal Medicine, Faculty of Medicine, Tottori University, Yonago, JPN; 2 Division of Dermatology, Department of Medicine of Sensory and Motor Organs, Faculty of Medicine, Tottori University, Yonago, JPN

**Keywords:** covid-19, erythema nodosum, fasciitis, sars-cov-2, vaccine

## Abstract

Vaccines play a crucial role in preventing various infectious diseases, and their safety has been well established, with adverse effects generally being mild and transient. However, in rare instances, serious adverse events, including autoimmune diseases, have been reported following vaccination. Among these conditions, fasciitis is extremely rare. To our knowledge, the present report describes the first documented case of fasciitis occurring in combination with erythema nodosum after vaccination against severe acute respiratory syndrome coronavirus 2 (SARS-CoV-2). A 29-year-old Japanese woman presented with bilateral lower leg swelling, myalgia, ankle arthralgia, and transient painful erythema 15 days after vaccination. The erythema was diagnosed as erythema nodosum by skin biopsy and resolved spontaneously. Physical examination revealed swelling and tenderness of the gastrocnemius muscle, without skin sclerosis. Eosinophilia was not observed. Magnetic resonance imaging (MRI) demonstrated high signal intensity in the fascia and gastrocnemius muscles bilaterally. Creatine kinase levels and electromyography findings were normal, and myositis-specific autoantibody tests were negative. The patient was treated with 15 mg/day prednisolone, which led to symptomatic improvement. After discontinuation of glucocorticoids, swelling and tenderness of the gastrocnemius muscle worsened; however, the abnormal MRI findings subsequently resolved, and glucocorticoids were ultimately discontinued. Our review of previously reported cases of fasciitis following SARS-CoV-2 vaccination indicated favourable treatment responses in all patients, regardless of treatment with glucocorticoid monotherapy, combination immunosuppressive therapy, or no treatment. Although rare, clinicians should be aware of the possibility of post-vaccination fasciitis.

## Introduction

Severe acute respiratory syndrome coronavirus 2 (SARS-CoV-2) was first identified in December 2019 in Wuhan. Subsequently, coronavirus disease 2019 (COVID-19), caused by SARS-CoV-2 infection, spread worldwide, leading to a pandemic [1]. In response to this pandemic, the development of vaccines progressed rapidly, and SARS-CoV-2 vaccines, approved in December 2020, have played a crucial role in preventing infection and reducing the severity of disease [2]. Alongside advancements in COVID-19 treatments and therapeutic approaches, these vaccines have significantly contributed to resolving the pandemic. Unlike conventional inactivated or live attenuated vaccines, COVID-19 vaccination primarily relies on novel mRNA-based vaccines. As with other vaccines, adverse reactions to mRNA vaccines occur at a certain frequency; however, most reactions are mild and self-limiting. The efficacy and safety of mRNA vaccines have been well established [2], although rare serious adverse events such as myocarditis have also been reported [3,4].

Fasciitis encompasses a spectrum of conditions, including necrotising fasciitis caused by bacterial infection; nodular fasciitis classified as a benign soft tissue tumour; eosinophilic fasciitis, which is presumed to result from autoimmune mechanisms. Eosinophilic fasciitis is associated with several triggering factors, the most common being intense physical exertion. Other reported triggers include malignancy, trauma, medication use, and infection. Clinically, this condition typically presents as symmetrical, circumferential swelling and induration of the forearms or lower legs, often accompanied by erythema and pain in the early stages. As the disease progresses, the overlying skin may exhibit uneven texture and deep fibrosis. A slight male predominance is observed, with individuals aged 20-60 years being commonly affected. Oral glucocorticoids are the mainstay of treatment, and patients generally respond well [5].

Erythema nodosum is characterized by indurated, erythematous nodules under the skin, which are typically painful and predominantly affect the lower legs. Histopathologically, it is classified as septal panniculitis. It is considered to result from a hypersensitivity reaction to various underlying diseases or medications. Treatment consists mainly of rest and eliminating the underlying cause. When an associated disease is present, managing that condition is essential. While cases of fasciitis or erythema nodosum developing after vaccination have been reported, to our knowledge, there have been no previous reports describing the simultaneous occurrence of fasciitis and erythema nodosum following vaccination.

We report a case of fasciitis and erythema nodosum that developed following the administration of a SARS-CoV-2 mRNA vaccine.

## Case presentation

**Figure 1:**
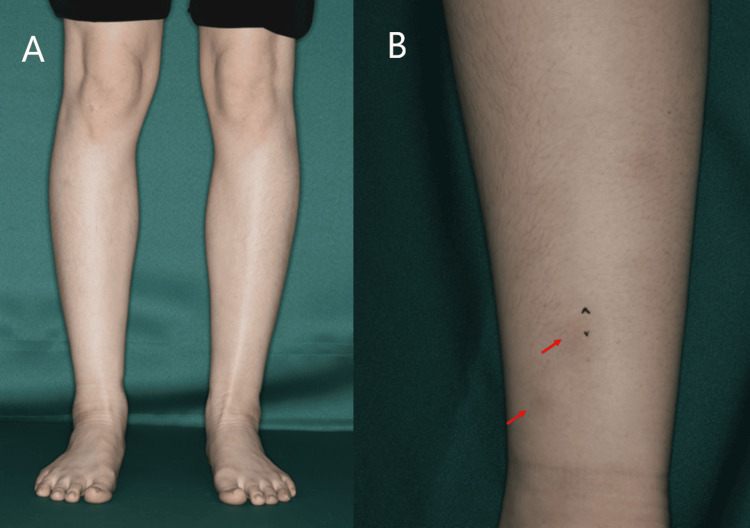
The patient's lower leg and erythema (A) Oedematous swelling of bilateral lower legs. (B) Posterior view of the right lower leg and ankle. Erythema is observed on the distal lower leg (arrow). The black mark indicates the site where the skin biopsy was performed.

A 29-year-old Japanese woman with a medical history of paediatric asthma and somatisation disorder presented with bilateral lower leg swelling and painful erythema, myalgia, and ankle arthralgia (Figure 1).

**Figure 2:**
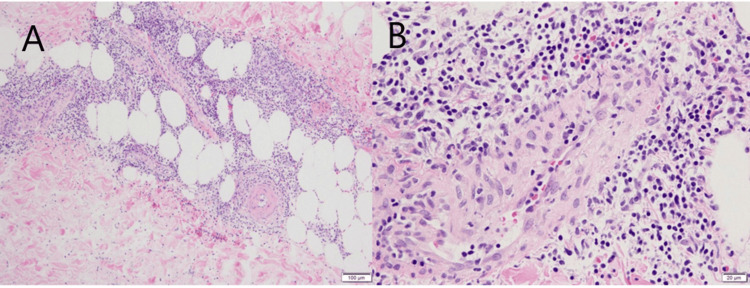
The histopathological findings of the lower leg erythema (A) Subcutaneous panniculitis that predominantly affects the septal areas (Hematoxylin and eosin, x10). (B) Perivascular inflammatory cell infiltration, primarily consisting of lymphocytes, was demonstrated in a subcutaneous septal area (Hematoxylin and eosin, x40).

At the time of presentation, the patient was taking oral etizolam and a drospirenone/ethinylestradiol betadex preparation for 11 years. She had no history of COVID-19 infection. These symptoms appeared 15 days after receiving the first dose of the SARS-CoV-2 vaccine (BNT162b2, Pfizer/BioNTech). Skin biopsy of the erythema on the right lower leg revealed perivascular infiltration of predominantly lymphocytic inflammatory cells in the dermis, along with septal lymphocytic infiltration and extravasation of red blood cells in the subcutis. Despite the presence of perivascular inflammatory cell infiltration, no histopathological features indicative of vasculitis were observed (Figure 2).

Based on these findings, a diagnosis of erythema nodosum (EN) was made. The erythema resolved spontaneously; however, pain in the bilateral lower legs and ankle joints persisted, and the swelling gradually worsened. The patient was subsequently referred to our department. The patient presented without any history of rheumatic or neuromuscular disease, trauma, or strenuous exercise.

Physical examination

Physical examination revealed mild pitting oedema in both lower legs, with swelling and tenderness of the gastrocnemius muscles, and tenderness around the ankles. The patient was afebrile and exhibited neither skin rash (erythema) nor joint swelling. There was no tenderness at the entheses, including the Achilles tendon. No signs of skin induration or groove sign were observed.

Investigations

**Figure 3:**
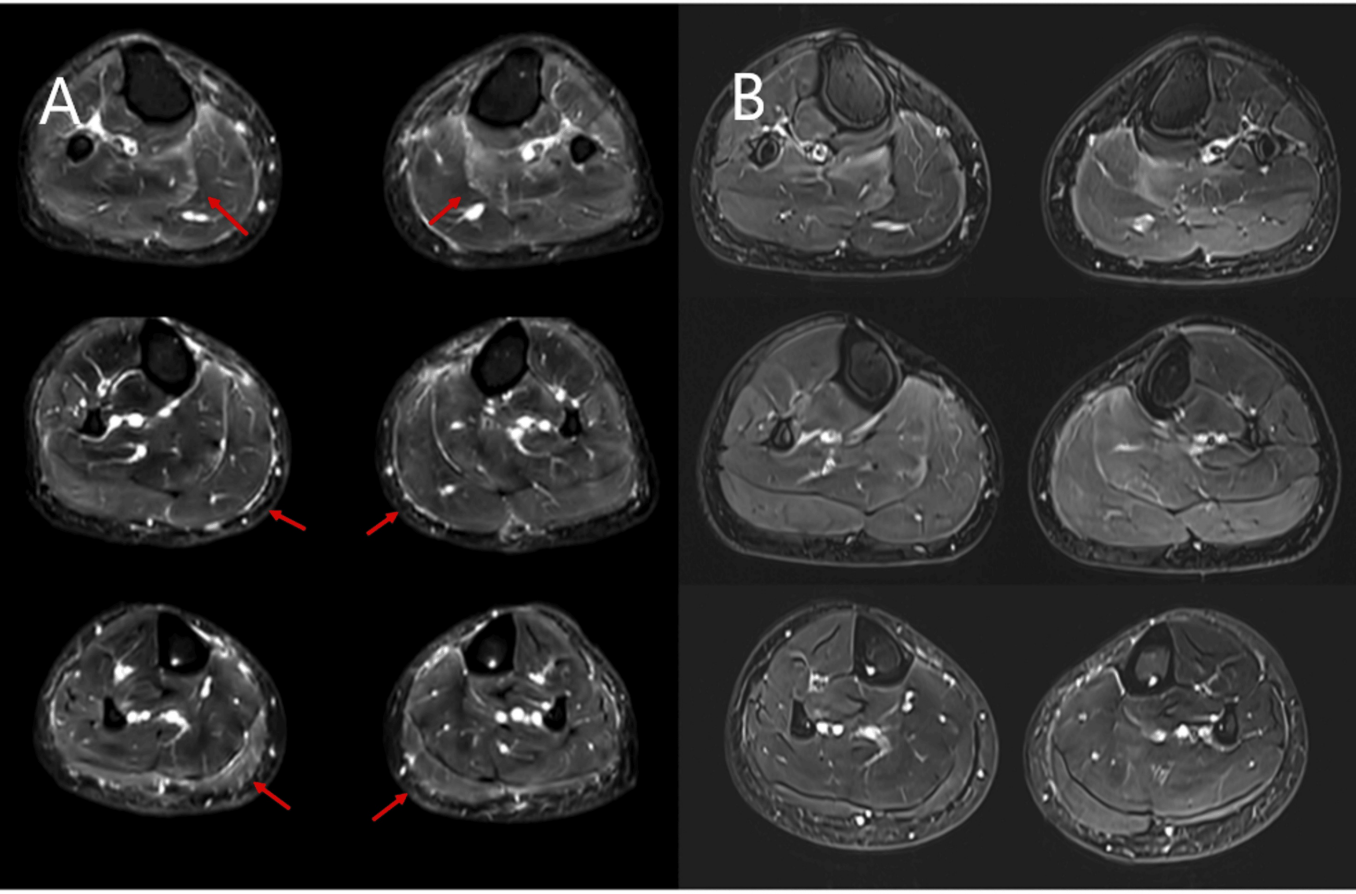
Bilateral lower legs on MRI before and after treatment Short Tau Inversion Recovery (STIR) images of the bilateral lower legs on MRI demonstrate high signal intensity in both gastrocnemius muscles before treatment, with particularly pronounced signals along the fascia (arrows). After treatment, these high-signal areas in the gastrocnemius muscles and fascia resolved. (A) Before treatment; (B) after treatment.

Blood tests revealed C-reactive protein (CRP) levels of <0.01 mg/dL, creatine kinase (CK) levels of 53 U/L, aldolase levels of 4.6 U/L, white blood cell count of 5,500/μL, eosinophil count of 39/μL, and the presence of an antinuclear antibody at a titer of 1:80 (homogeneous and speckled pattern). Autoantibody testing showed the following: anti-cyclic citrullinated peptide antibody ≤0.6 U/mL, anti-double-strand DNA antibody ≤0.6 IU/mL, anti-U1-ribonucleoprotein antibody 3.3 U/mL (<10.0 U/mL), anti-aminoacyl tRNA synthetase antibody 5.2 index (<25.0 index), myeloperoxidase-antineutrophil cytoplasmic antibody (ANCA) ≤1.0 U/mL, and proteinase 3-ANCA ≤1.0 U/mL. Magnetic resonance imaging (MRI) of both lower extremity muscles revealed high signal intensity in the gastrocnemius muscles on Short Tau Inversion Recovery (STIR) images, with particularly prominent signals in the fascia (Figure 3A). Needle electromyography revealed no abnormal findings. Although muscle biopsy was recommended, the patient declined, and the procedure was not performed. Eosinophilic fasciitis was excluded based on the absence of cutaneous manifestations, skin histopathology, and normal eosinophil counts. Based on these results, fasciitis was diagnosed. Given the onset of symptoms following SARS-CoV-2 vaccination, a diagnosis of vaccine-related fasciitis was made.

Treatment course and outcome

The patient was initially treated with nonsteroidal anti-inflammatory drugs (NSAIDs); however, no symptomatic improvement was observed. Subsequently, prednisolone (PSL) was initiated at 15 mg/day, resulting in rapid clinical improvement of gastrocnemius muscle swelling and tenderness, as well as ankle tenderness. PSL was gradually tapered and temporarily discontinued after eight months; nevertheless, gastrocnemius swelling and tenderness worsened, without elevation of CK or CRP. PSL was reintroduced at 3 mg/day two months after discontinuation. As the pain persisted, a follow-up MRI of the lower limbs was performed, revealing resolution of the previously noted high signal intensity in the gastrocnemius muscle and fascia (Figure 3B).

As the findings suggested an improvement in fasciitis, PSL was tapered and discontinued, and adjunctive analgesics were introduced. No relapse was observed over the subsequent six months. Thereafter, the need for adjunctive analgesics decreased.

## Discussion

In dermatomyositis, inflammatory myopathy, along with various environmental factors and genetic predisposition, has been implicated in disease onset. Known triggers for disease development or exacerbation include medications such as statins, vaccines, infections, ultraviolet exposure, cigarette smoke, chemical exposures, and malignancies [6]. Among these, viral infections have been suggested as potential triggers of various autoimmune diseases, including systemic lupus erythematosus, ANCA-associated vasculitis, and neuroimmunological disorders [7-9]. Moreover, there have been reports of autoimmune diseases, including vasculitis and systemic lupus erythematosus, developing after mRNA vaccination [10,11]. In the context of autoimmune diseases associated with viral infections or vaccination, the pathogenesis of inflammatory muscle diseases is thought to involve cross-reactivity between major epitopes of viral spike proteins and autoantigens related to muscle inflammation.

Although several cases of inflammatory myopathies, such as polymyositis and dermatomyositis, have been reported following SARS-CoV-2 vaccination [12,13], fasciitis as a postvaccination manifestation remains rare. A total of five cases, including the present case, have been identified [14-17]; these are summarized in Table 1. The age of the patients ranged from 20 to 70 years; three were female, and four cases involved mRNA vaccines. Three patients developed symptoms after the first vaccine dose, and four experienced symptom onset within 15 days of vaccination. In two cases, the sites of fasciitis involved both the upper and lower limbs, and in three cases, only the lower limbs were affected. One patient improved without treatment; however, the others needed glucocorticoid therapy, with two requiring immunosuppressive agents. All patients improved, which suggested that fasciitis generally responds well to immunosuppressive treatments such as glucocorticoids. The clinical features, including patient background and treatment responses, resembled those seen in patients with eosinophilic fasciitis triggered by other factors.

**Table 1:** A review of reported cases of fasciitis following SARS-CoV-2 vaccination Abbreviations: SARS-CoV-2, severe acute respiratory syndrome coronavirus 2; GC, glucocorticoid; MMF, mycophenolate mofetil; MTX, methotrexate

Case	Age	Sex	Type of vaccine	Time to onset	Type of fasciitis	Site of fasciitis	Treatment	Outcome
Case 1 [14]	72	Female	mRNA	2nd dose 4 weeks	Eosinophilic fasciitis	Upper and lower limbs	GC+MMF	improved
Case 2 [15]	28	Female	mRNA	1st dose 5 days	Fasciitis + rhabdomyolysis	Lower Limbs	GC pulse, GC	improved
Case 3 [16]	55	Male	recombinant	1st dose 1 week	Eosinophilic fasciitis	Upper and lower limbs	GC pulse, GC+MTX	improved
Case 4 [17]	39	Male	mRNA	2nd dose 6 days	Unknown	Lower Limbs	No treatment	improved
This Case	29	Female	mRNA	1st dose 15 days	Fasciitis + myositis	Lower Limbs	GC	improved

In the present case, EN developed concurrently with fasciitis. Although there have been reports of EN developing after SARS-CoV-2 mRNA vaccination, the incidence is considered extremely low [18]. The patient had been taking a low-dose estrogen-progestin preparation for a long period. Although long-term use of this drug can cause EN, the EN resolved spontaneously without discontinuation of the medication, and there was no recurrence during long-term follow-up. Therefore, we concluded that the EN in this case was not drug-induced, but rather triggered by the vaccination. Additionally, there have been no reported cases of EN and fasciitis occurring simultaneously following vaccination. Kobayashi et al. summarized 20 cases of EN after COVID-19 vaccination, 13 of which (65%) occurred after the first dose. Among 10 patients who underwent skin biopsy, five showed granulomatous changes characterized by histiocytes and multinucleated giant cells infiltrating the dermis, described as distinctive findings [19]. In our case, such findings were absent; however, unlike typical EN, there was prominent perivascular lymphocytic infiltration without evidence of vasculitis. The mechanism underlying EN after vaccination remains unclear, but it is generally regarded as a hypersensitivity reaction to diverse antigenic stimuli. In the post-vaccination setting, a delayed-type hypersensitivity reaction to vaccine components has been postulated as a potential mechanism.

The mechanism whereby fasciitis develops after SARS-CoV-2 vaccination remains unclear. In the present case, fasciitis developed bilaterally on the lower legs, distant from the vaccination site on the upper arm. Therefore, it was hypothesised that the condition was not caused by local stimulation or inflammatory responses at the injection site but rather by a systemic autoimmune mechanism, such as cross-reactivity with autoantigens. In this case, both fasciitis and EN developed simultaneously after vaccination. Since both conditions are considered to result from autoimmune mechanisms such as hypersensitivity reactions, their concurrent involvement in the lower legs is of particular interest. As EN resolved earlier than fasciitis and the histopathological findings differed from those of typical EN, it is plausible that the autoimmune response underlying fasciitis may have extended to the skin and contributed to the development of EN.

Currently, there is no clear evidence regarding revaccination in individuals who experience autoimmune-related adverse events after vaccination. However, the Centers for Disease Control and Prevention (CDC) recommends that individuals who develop myocarditis or pericarditis following vaccination should generally avoid further doses [20]. In general, revaccination should be avoided in patients who experience severe adverse events. However, in cases like this one, where the adverse event was not life-threatening or caused irreversible organ damage, the decision to revaccinate should be made on a case-by-case basis. This requires a careful assessment of the risks and benefits, as well as shared decision-making with the patient.

## Conclusions

We report the first case of bilateral lower leg fasciitis and erythema nodosum that developed following SARS-CoV-2 mRNA vaccination. Fasciitis and erythema nodosum have only been reported in a small number of cases following vaccination. To our knowledge, no previous reports have described the occurrence of both conditions simultaneously. When persistent muscle pain arises in areas distant from the injection site after SARS-CoV-2 vaccination, fasciitis, though rare, may be considered as a potential differential diagnosis in similar clinical contexts.
